# Microhydration Effects on Hemibonds in [H_2_O–X]^+^–(H_2_O)_*n*_ (X = O_2_ and CS_2_; *n* = 0–2): Infrared Spectroscopic Characterization toward Understanding Charge-Resonance Interactions in Aqueous Environments

**DOI:** 10.1021/acs.jpclett.6c00621

**Published:** 2026-03-23

**Authors:** Tatsuki Hosoda, Asuka Fujii

**Affiliations:** Department of Chemistry, Graduate School of Science, Tohoku University, Sendai 980-8578, Japan

## Abstract

The hemibond, a nonclassical covalent interaction arising from charge-resonance between a radical and a neutral molecule, represents a distinctive bonding motif in open-shell systems. Its role has been widely discussed in radical reactions, radiation chemistry, and related biochemical processes. While hemibonds involving water molecules have garnered considerable interest, it remains unclear whether these interactions can persist under bulk solvation conditions. Here, we investigate hemibond formation in gas-phase [H_2_O–X]^+^ clusters and examine the structural evolution upon microhydration. Infrared photodissociation spectroscopy of [H_2_O–X]^+^–(H_2_O)_*n*_ (X = O_2_ and CS_2_; *n* = 0–2) reveals that the hemibonded structure of [H_2_O–X]^+^ persists during microhydration. These results elucidate the interplay between charge-resonance and charge-(induced) dipole interactions that govern hemibond stability and suggest that certain molecules may retain the ability to form stable hemibonds with water even in aqueous environments.

## Graphical abstract

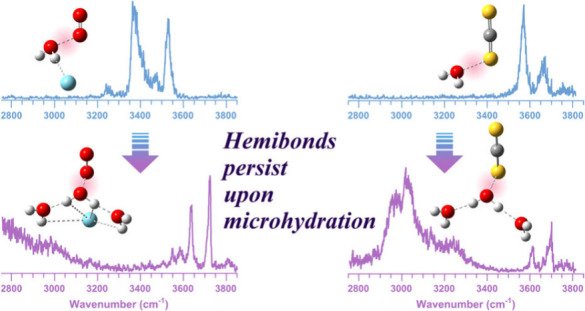

Radical species challenge conventional views of chemical bonding. In open-shell systems, intermolecular interactions can emerge that are not adequately described by traditional two-electron covalent bonds, prompting a re-examination of fundamental bonding concepts. Among these unconventional motifs, the hemibond—a two-center three-electron (2c-3e) bond—has attracted sustained theoretical and experimental interest.^1–16^ In a hemibond, the interaction between two nonbonding orbitals of a radical (or radical cation) and a closed shell molecule results in bonding and antibonding combinations; the bonding orbital is doubly occupied and the antibonding orbital singly occupied, leading to an effective bond order of 1/2. This electronic structure originates from intermolecular charge-resonance interactions, in which an unpaired electron is partially delocalized between nearly degenerate electronic configurations. The hemibond has long been discussed in radiation chemistry, particularly in sulfur-containing systems,^1–7,10–15,17–28^ and its potential relevance to biochemical processes is increasingly recognized.^29–36^ Water occupies a uniquely important position in this context. Because water is the dominant component of biological and environmental systems, understanding how hemibonds involving water behave in condensed phases is of fundamental importance. Yet, despite decades of study on the water radical cation,^37–79^ the presence and roles of hemibonds in aqueous environments remain largely unresolved. In bulk water, strong solvation and proton-transfer dynamics obscure direct structural identification, making it difficult to disentangle charge-resonance stabilization from competing hydrogen-bonding and proton-transfer motifs.

Gas-phase cluster spectroscopy provides a molecular-level platform to overcome this limitation. Isolation of well-defined clusters enables systematic introduction of solvent molecules and monitoring of structural evolution under controlled conditions. In particular, the stepwise addition of a small number of water molecules, referred to as microhydration, offers a unique opportunity to investigate the first microscopic steps toward the aqueous phase. In this sense, microhydrated clusters serve as molecular bridges between gas-phase complexes and the aqueous environment. Before addressing solvation effects, however, it is essential to establish the intrinsic electronic factors governing hemibond formation in [H_2_O–X]^+^, where X denotes the molecular partner of H_2_O. Recent gas-phase studies have revealed that hemibonded structures emerge when the ionization potential (IP) of X is close to that of H_2_O (e.g., N_2_O, Kr, and CO).^65,76,77^ As the IP difference (ΔIP) between X and H_2_O decreases, the energetic mismatch between interacting nonbonding orbitals is reduced, promoting electron delocalization and strengthening charge-resonance interactions.^14,18^ Nevertheless, even when ΔIP is zero, as exemplified by water dimer cation (H_2_O)_2_^+^, proton transfer to form H_3_O^+^–OH is favored over hemibond formation.^37–55^ This behavior reflects not only the intrinsically strong acidity of H_2_O^+^ but also the high proton affinity (PA) of neutral H_2_O, both of which strongly stabilize the proton-transferred structure. These findings indicate that the formation of hemibonds involving water is governed by the interplay between IP and PA under competition with proton transfer.

To date, most studies of hemibonds involving water have primarily focused on the water radical cation H_2_O^+^, which plays a key role in the biology of various radiation-related processes, and thus have mainly examined systems where X possesses an IP higher than that of H_2_O and therefore the charge is more distributed in the water moiety. In these systems, the progression of microhydration often triggers proton transfer from H_2_O^+^, leading to the disruption of the hemibond between water and X. In contrast, hemibonded structures formed between H_2_O and molecules with slightly lower IPs than H_2_O remain scarcely explored, and direct gas-phase spectroscopic evidence is lacking.^80,81^ When the IP of X is lower than that of H_2_O, electrostatic repulsion between the positively charged X and the partially positively charged hydrogen atoms of H_2_O can suppress competition with hydrogen-bonded or proton-transferred structures in [H_2_O–X]^+^. Such systems may therefore permit the formation of hemibonds involving water and serve as model systems for probing their response to solvation.

In this work, we investigate [H_2_O–X]^+^ (where X = O_2_ and CS_2_, which have IPs of 12.1 and 10.1 eV, respectively, both slightly lower than, yet close to, that of H_2_O (12.6 eV)),^82^ and systematically examine their microhydrated clusters, [H_2_O–X]^+^–(H_2_O)_*n*_ (*n* = 1 and 2), using infrared photodissociation (IRPD) spectroscopy. By incrementally adding water molecules, we directly assess how microhydration influences hemibond stability. Although hydration-induced destabilization of hemibonds has been reported in several systems,^23–25,79^ whether hemibonds involving water can persist under increasing solvation remains an open question directly relevant to aqueous environments. The present study provides molecular-level insight into the conditions under which hemibonds involving water can survive and establishes a conceptual framework that connects isolated gas-phase clusters to solvated environments, thereby advancing our understanding of radical interactions in aqueous systems.

A schematic diagram of the experimental apparatus and experimental details are provided in the Supporting Information (SI). In addition to the clusters described above, tagging methods^83,84^ were employed: Ar-tagging for O_2_-containing clusters and N_2_-tagging for CS_2_-containing clusters. Their low dissociation energies relative to the IR photon energy ensure reliable dissociation spectroscopy, and the tagging also provides a cooling effect to access the most stable structures. For CS_2_, N_2_-tagging was employed because Ar-tagged clusters did not yield sufficient signal intensity. In the following, we focus on the Ar-tagged [H_2_O–O_2_]^+^–(H_2_O)_*n*_ clusters (*n* = 0–2), for which more definitive spectral assignments are possible. For [H_2_O–CS_2_]^+^–(H_2_O)_*n*_ (*n* = 0–2), the bare clusters (clusters without a tagging species) provided sufficiently clear insights. Results for other species are summarized in the SI. The observed spectral features were analyzed by comparison with quantum chemical calculations performed using the Gaussian 16 program package;^85^ computational details are also described in the SI.

Figures 1(a) and (b) show the observed IR spectra of the [H_2_O–O_2_]^+^–Ar and [H_2_O–CS_2_]^+^, respectively, together with the optimized stable structures and their calculated spectra at the CCSD/aug-cc-pVDZ level. For the bare clusters, only a single stable structure was found in each case. In these structures, molecule X (i.e., O_2_ or CS_2_) binds to H_2_O via a hemibond, arising from the overlap of lone-pair orbitals; these structures are classified as type-I (i.e., hemibonded type). No stable hydrogen-bonded isomers, in which molecule X attaches to the hydrogen atom end of the OH group, were obtained, as anticipated above. Several stable tagged isomers were identified depending on the tag binding site; only the most stable isomer is shown in the figure. The label “W1” denotes that the system contains one water molecule, and the molecules listed in parentheses indicate constituents other than H_2_O in the cluster.

**Figure 1:**
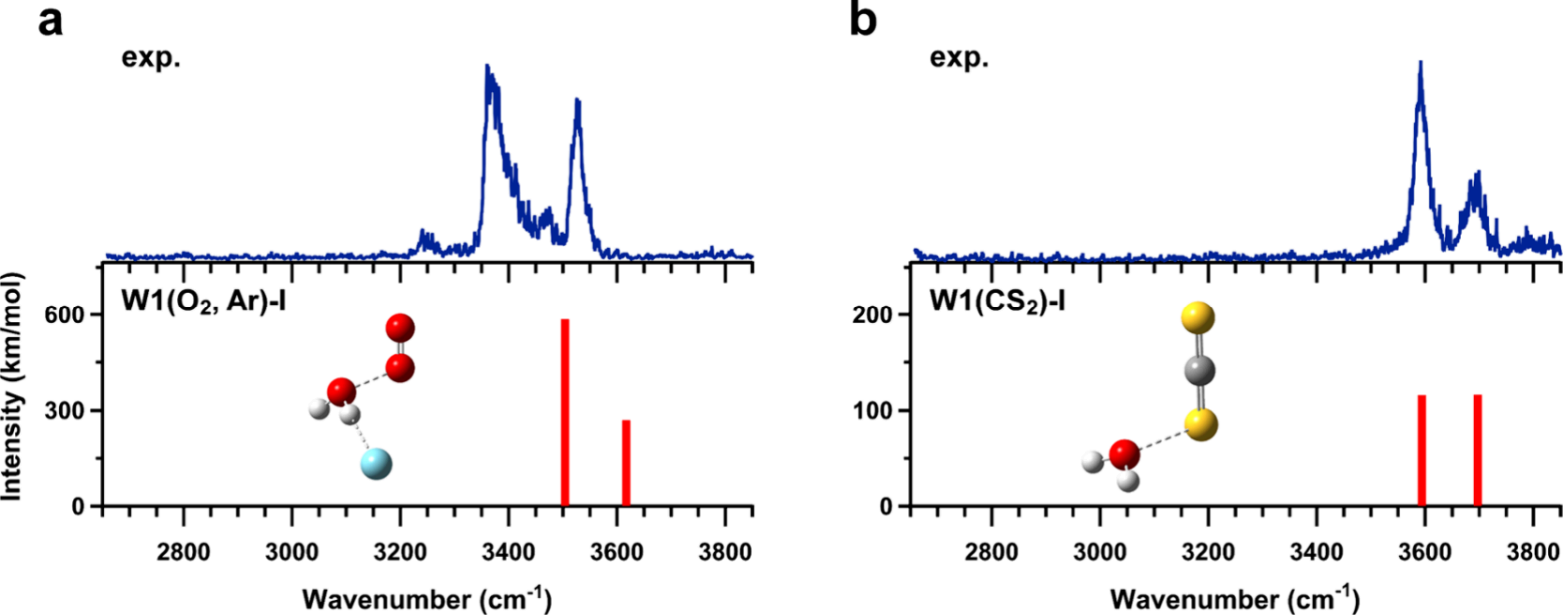
Comparisons of the observed IR spectra of (a) [H_2_O–O_2_]^+^–Ar and (b) [H_2_O–CS_2_]^+^ with the spectra calculated at the CCSD/aug-cc-pVDZ level of theory. The calculated vibrational frequencies are scaled by a factor of 0.951. The corresponding optimized structures are also shown.

In the observed spectrum of [H_2_O–O_2_]^+^–Ar, two prominent bands appear at approximately 3400 and 3530 cm^–1^. These bands are assigned to the Ar-bound OH stretching and the free OH stretching vibration, respectively, as calculated for W1­(O_2_, Ar)-I. In addition, weak features observed at 3250 and 3470 cm^–1^ are assigned to the bending overtone of H_2_O and a combination band involving the Ar-bound OH stretching and the intermolecular stretching vibration, respectively. These spectral features are similar to those previously reported for the [H_2_O–N_2_O]^+^–Ar, supporting the assignment of the type-I structure.^77^ In the observed spectrum of [H_2_O–CS_2_]^+^, two distinct bands are detected at approximately 3600 and 3700 cm^–1^. These bands are assigned to the symmetric and antisymmetric OH stretching modes (ν_1_ and ν_3_) of the H_2_O moiety, as calculated for the W1­(CS_2_)-I structure. Notably, the bands of [H_2_O–O_2_]^+^–Ar appear at lower frequencies than those of [H_2_O–CS_2_]^+^. This arises from the much lower charge localization on the water moiety in [H_2_O–CS_2_]^+^, as discussed later.

Figures 2(a) and (b) show comparisons between observed and calculated IR spectra of [H_2_O–O_2_]^+^–H_2_O–Ar and [H_2_O–O_2_]^+^–(H_2_O)_2_–Ar, respectively. The numbers given in parentheses indicate zero-point energy (ZPE) corrected relative energies in kJ/mol, calculated at the CCSD­(T)/aug-cc-pVTZ//CCSD/aug-cc-pVDZ level. For these systems, three types of stable structures were obtained: type-I, as described above; type-II, in which two water molecules bind to O_2_ from the opposite side (i.e., multiple hemibonded type); and type-III, in which proton transfer between water molecules results in the formation of an H_3_O^+^ core hydrogen-bonded to O_2_ (i.e., proton-transferred type). In Figure 2(a), four prominent bands are experimentally observed: a broad band around 2800 cm^–1^, a strong band near 3430 cm^–1^, and two bands above 3600 cm^–1^. According to the calculated spectrum for the most stable W2­(O_2_, Ar)-I, these bands are assigned to the OH stretching vibration of central H_2_O hydrogen-bonded to another H_2_O, Ar-bound OH stretching vibration, and the ν_1_ and ν_3_ modes of terminal H_2_O. All the bands in the observed spectrum can be reasonably assigned to W2­(O_2_, Ar)-I; furthermore, because W2­(O_2_, Ar)-II and W2­(O_2_, Ar)-III lie much higher in relative energy, their contributions are expected to be negligible. Therefore, the observed isomer is concluded to be W2­(O_2_, Ar)-I. In Figure 2(b), three prominent bands are observed in the experimental spectrum of [H_2_O–O_2_]^+^–(H_2_O)_2_–Ar. The band around 2700 cm^–1^ is assigned to the OH stretching vibration of the hydrogen-bonded OH group, while the bands at about 3640 and 3730 cm^–1^ are assigned to the ν_1_ and ν_3_ bands of neutral H_2_O moieties as calculated for W3­(O_2_, Ar)-I. In addition, weak bands are also observed in the 3550–3600 cm^–1^ region. These bands are likely attributable to the contribution of W3­(O_2_, Ar)-III, corresponding to the O_2_-bound OH stretching and OH radical stretching vibrations. Based on the transition intensity ratio between the calculated spectra of W3­(O_2_, Ar)-I and W3­(O_2_, Ar)-III, the population of W3­(O_2_, Ar)-III is estimated to be less than 10%. Therefore, the observed structures are concluded to consist predominantly of W3­(O_2,_ Ar)-I, with W3­(O_2_, Ar)-III coexisting as a minor component. IR spectra of bare [H_2_O–O_2_]^+^–(H_2_O)_*n*_ (*n* = 0–2) were also measured. A comparison between the experimental and calculated spectra is provided in Figure S3 in the SI. In all cases, the results are parallel to those obtained from the Ar-tagged spectra, leading to the same conclusions regarding the isomeric structures.

**Figure 2:**
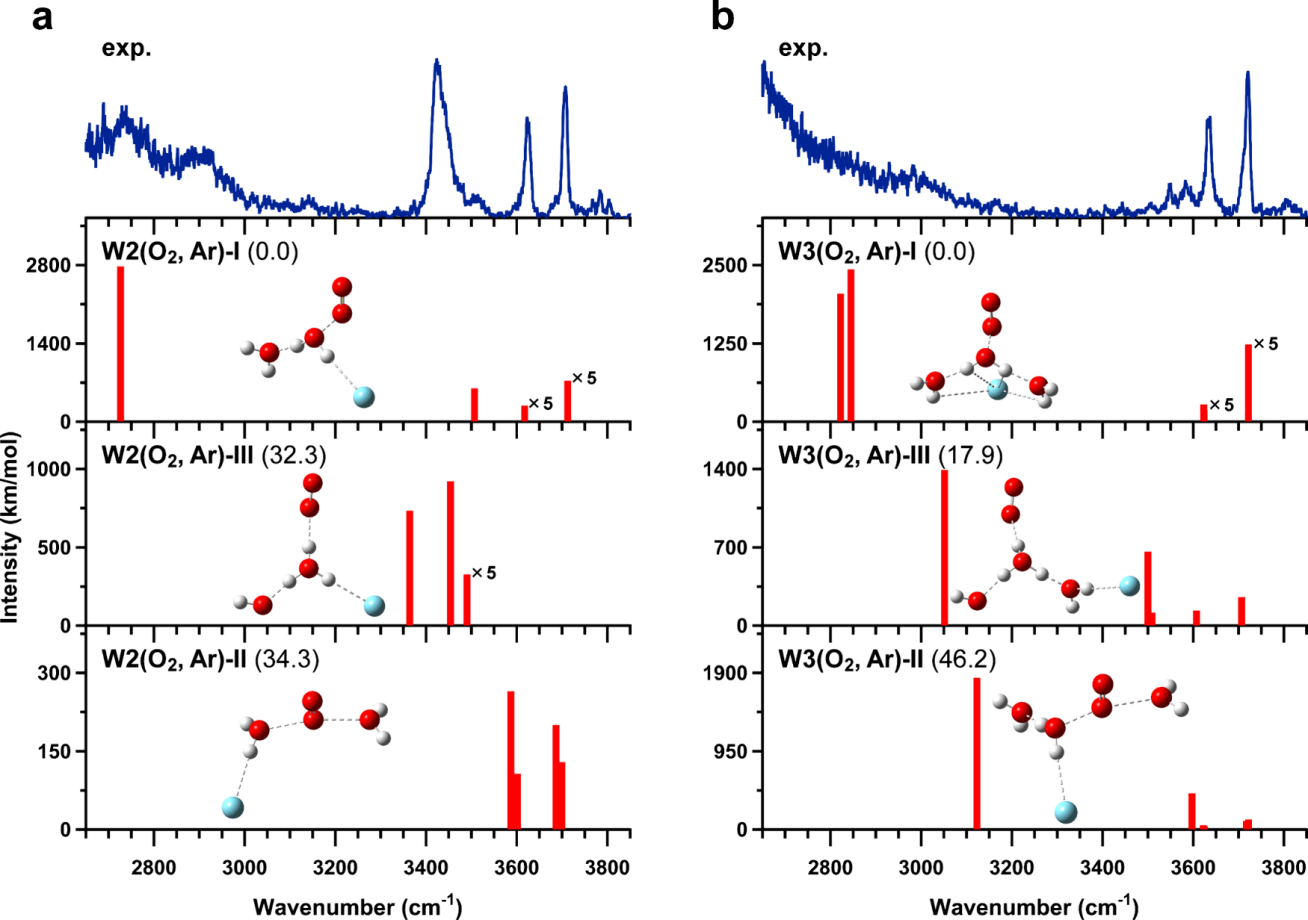
Comparisons of the observed IR spectra of (a) [H_2_O–O_2_]^+^–H_2_O–Ar and (b) [H_2_O–O_2_]^+^–(H_2_O)_2_–Ar with the calculated spectra of three stable structures obtained at the CCSD/aug-cc-pVDZ level of theory. The calculated spectra are scaled by a factor of 0.951. The values given in parentheses indicate relative energies (in kJ/mol), obtained by adding ZPE corrections calculated at the CCSD/aug-cc-pVDZ level to the electronic energies computed at the CCSD­(T)/aug-cc-pVTZ level.

Figures 3(a) and (b) show comparisons between observed and calculated IR spectra of [H_2_O–CS_2_]^+^–H_2_O and [H_2_O–CS_2_]^+^–(H_2_O)_2_, respectively. No stable type-III structures were found in the calculations. In Figure 3(a), the experimental spectrum of [H_2_O–CS_2_]^+^–H_2_O is well reproduced by the calculated spectrum of W2­(CS_2_)-I. The broad band around 3000 cm^–1^ is assigned to the OH stretching vibration of the hydrogen-bonded OH group and the band at 3730 cm^–1^ is assigned to the ν_3_ mode of terminal H_2_O. In addition, a strong band is observed around 3630 cm^–1^. In the calculated spectrum of W2­(CS_2_)-I, the ν_1_ mode of terminal H_2_O is predicted at 3629 cm^–1^, while the dangling OH stretching vibration of central H_2_O is also predicted at 3636 cm^–1^. The observed band around 3630 cm^–1^ is therefore attributed to overlapping contributions from these two vibrational modes. This assignment was confirmed by measurements of N_2_-tagged [H_2_O–CS_2_]^+^–H_2_O clusters, in which coordination of N_2_ to the dangling OH group of central H_2_O induces a red shift of the corresponding band, allowing the N_2_-bound OH stretching vibration and the ν_1_ mode of terminal H_2_O to be separately observed. These results of the N_2_-tagged measurements are summarized in Figure S4 in the SI. The calculated spectrum of W2­(CS_2_)-II also shows good agreement with the experimental band positions in the free OH stretching region. Thus, although a minor contribution from W2­(CS_2_)-II cannot be completely excluded, its contribution is expected to be extremely limited due to its significantly higher relative energy. Therefore, the observed isomer is concluded to be W2­(CS_2_)-I. In Figure 3(b), three remarkable bands are observed in the experimental spectrum: a broad band around 3000 cm^–1^ and two bands above 3600 cm^–1^. By comparison with the calculated spectrum of W3­(CS_2_)-I, these bands are assigned to the hydrogen-bonded OH stretch and the ν_1_ and ν_3_ modes of neutral H_2_O, respectively. The contribution of W3­(CS_2_)-II is expected to be negligible due to its higher relative energy. Accordingly, the observed isomer is concluded to be W3­(CS_2_)-I.

Overall, these results demonstrate that, for systems in which the IP of molecule X is close to but lower than that of H_2_O, the type-I (hemibonded) structure remains intact upon microhydration by one or two water molecules. This behavior contrasts with that previously reported for the microhydration of [H_2_O–N_2_O]^+^, in which the hemibonded-type ion core switches to the proton-transferred type upon microhydration.^79^

**Figure 3:**
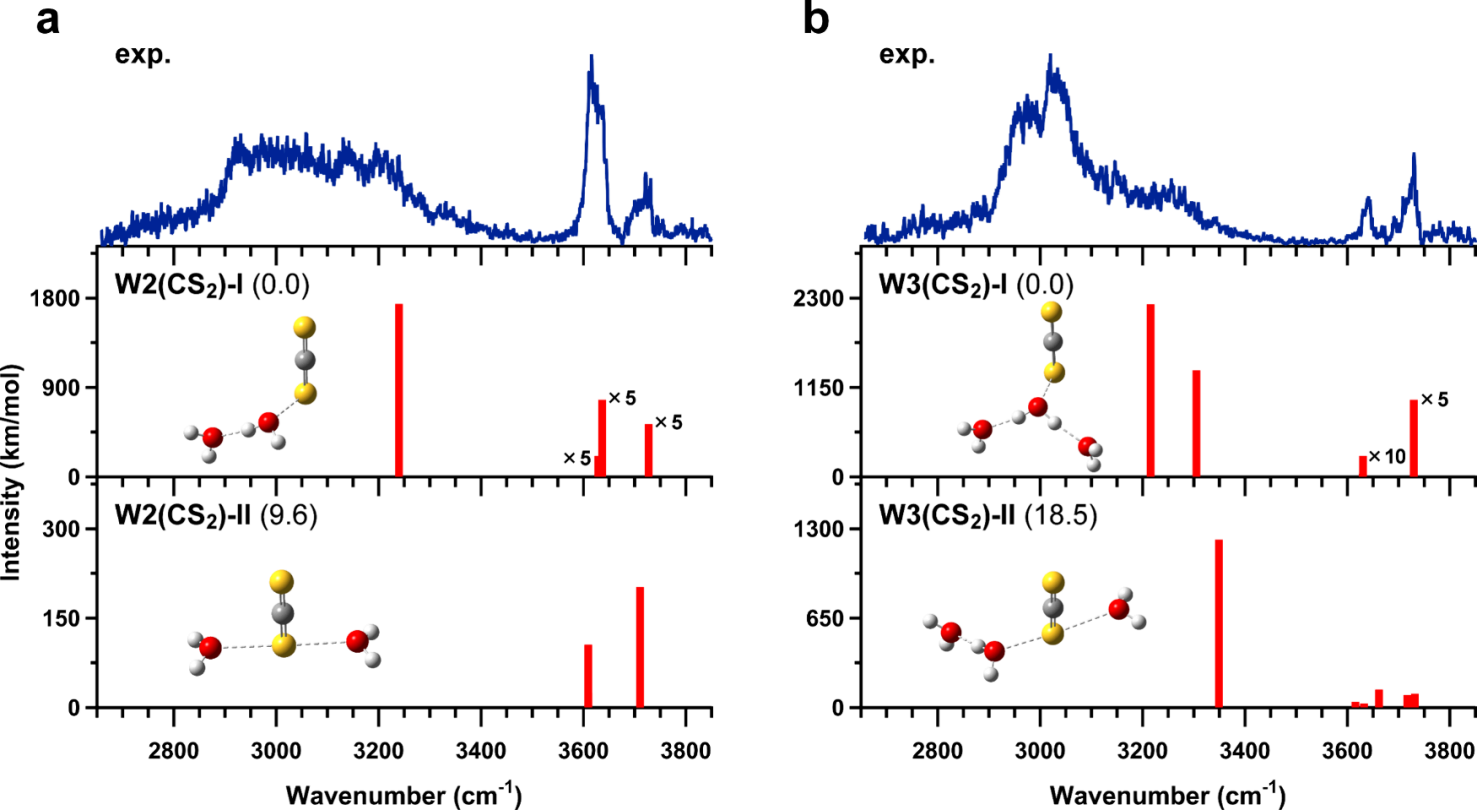
Comparisons of the observed IR spectra of (a) [H_2_O–CS_2_]^+^–H_2_O and (b) [H_2_O–CS_2_]^+^–(H_2_O)_2_ with the calculated spectra of two stable structures obtained at the CCSD/aug-cc-pVDZ level of theory. The calculated spectra are scaled by a factor of 0.951. The values given in parentheses indicate relative energies (in kJ/mol), obtained by adding ZPE corrections calculated at the CCSD/aug-cc-pVDZ level to the electronic energies computed at the CCSD­(T)/aug-cc-pVTZ level.

Figures 4(a)-(c) present the optimized structures of W1­(O_2_)-I, W2­(O_2_)-I, and W3­(O_2_)-I, together with the natural bond orbital (NBO) charges (labeled in black), spin density distributions, and binding energies between O_2_ and H_2_O (labeled in blue). Upon successive microhydration of [H_2_O–O_2_]^+^, the binding energy between O_2_ and H_2_O decreases monotonically, whereas the spin density becomes increasingly delocalized over O_2_ and H_2_O. At first glance, these trends appear contradictory, because enhanced spin delocalization is generally associated with a stronger hemibond contribution. This apparent inconsistency indicates that the binding interaction is not governed solely by charge-resonance effects (i.e., orbital interactions). To rationalize this behavior, it is necessary to consider contributions of nonorbital interactions in addition to charge-resonance interactions. In the present systems, such stabilization arises from two distinct components: charge-dipole (electrostatic) interactions between the positively charged X moiety and the permanent dipole moment of H_2_O, and charge-induced dipole (induction) interactions, in which the electric field generated by the localized positive charge on X polarizes the electron density of H_2_O, thereby inducing a dipole moment. Charge-resonance interactions are strengthened as the positive charge becomes increasingly delocalized over O_2_ and H_2_O, whereas the charge-(induced) dipole interactions are enhanced when the positive charge is more strongly localized on O_2_, resulting in a larger local electric field acting on H_2_O.

**Figure 4:**
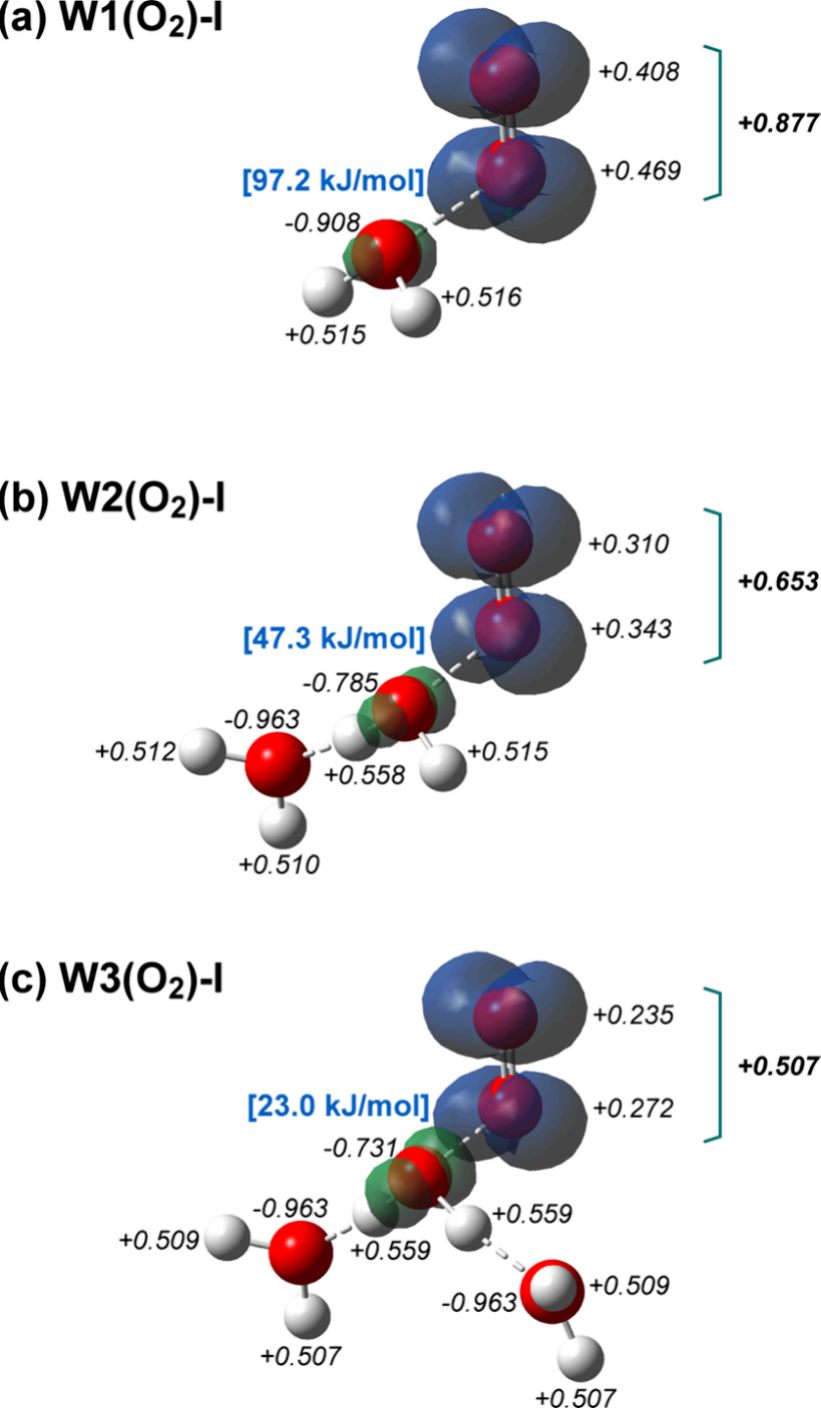
(a–c) Type-I structures of [H_2_O–O_2_]^+^–(H_2_O)_*n*_ (*n* = 0–2), together with the NBO charges (black), spin density distributions, and the binding energies of the O_2_–OH_2_ bond (blue). The NBO charges were calculated at the CCSD­(T)/aug-cc-pVTZ level. The spin densities were calculated at the CCSD/aug-cc-pVDZ level, and the density is plotted at an isosurface value of 0.004. The binding energies were evaluated from BSSE-corrected electronic energies at the CCSD­(T)/aug-cc-pVTZ level with ZPE corrections performed at the CCSD/aug-cc-pVDZ level.

In W1­(O_2_)-I, O_2_ retains most of the positive charge (+0.877), resulting in a weak charge-resonance interaction but a strong charge-(induced) dipole interaction. Upon addition of H_2_O to form W2­(O_2_)-I, partial charge transfer to water reduces the positive charge on O_2_ to +0.653, strengthening the charge-resonance while simultaneously weakening the charge-(induced) dipole contribution. Further microhydration to form W3­(O_2_)-I leads to an even lower positive charge on O_2_ (+0.507), further enhancing charge-resonance effects. However, because the reduction in charge-(induced) dipole stabilization is more pronounced, the overall binding energy of the O_2_–OH_2_ bond decreases with increasing microhydration. Thus, in the O_2_-containing clusters, microhydration enhances charge-resonance interactions but destabilizes the O_2_–OH_2_ bond by weakening nonorbital interactions. We should note that the total energy of the system is lowered by the formation of hydrogen bonds between H_2_O molecules. Therefore, this weakening of the O_2_–OH_2_ bond upon microhydration can be regarded as a type of anticooperative effect.

Figures 5(a)-(c) show the corresponding results for the W1­(CS_2_)-I, W2­(CS_2_)-I, and W3­(CS_2_)-I. In contrast to the O_2_-containing clusters, successive microhydration of [H_2_O–CS_2_]^+^ leads to a gradual increase in the binding energy between CS_2_ and H_2_O, accompanied by progressive delocalization of the unpaired electron. In W1­(CS_2_)-I, CS_2_ retains nearly all of the positive charge (+0.969), indicating that charge-(induced) dipole interactions are dominant in the CS_2_–OH_2_ bond, while charge-resonance interactions are weak. Despite the more pronounced localization of the positive charge on CS_2_, the binding energy of W1­(CS_2_)-I (50.5 kJ/mol) is significantly smaller than that of W1­(O_2_)-I (97.2 kJ/mol). This reduction is attributed to the larger atomic size of sulfur compared to oxygen, which weakens the charge-(induced) dipole interactions. The weaker interactions are consistent with the longer CS_2_–OH_2_ distance (2.60 Å) relative to the O_2_–OH_2_ distance (2.10 Å). Upon formation of W2­(CS_2_)-I, the positive charge on CS_2_ decreases only slightly to +0.910, indicating that charge transfer from CS_2_ to water is much smaller than in the O_2_-containing clusters. This difference originates from the lower IP of CS_2_ (10.1 eV) compared to that of O_2_ (12.1 eV), which suppresses charge transfer to H_2_O (12.6 eV).^82^ Consistent with this interpretation, the hydrogen-bonded OH stretching band in the experimental spectra appears at higher frequencies for the CS_2_-containing clusters than for the O_2_-containing clusters, indicating experimentally that the water moiety carries less positive charge in the CS_2_ systems. As a result, the charge-(induced) dipole interaction remains largely unchanged while the increased positive charge on the water moiety enhances charge-resonance interactions upon microhydration, leading to an increase in the binding energy. Further microhydration to form W3­(CS_2_)-I follows the same trend: the charge on CS_2_ decreases modestly to +0.828, the electrostatic contribution remains substantial, and the strengthened charge-resonance interaction dominates, resulting in a further increase in the binding energy.

**Figure 5:**
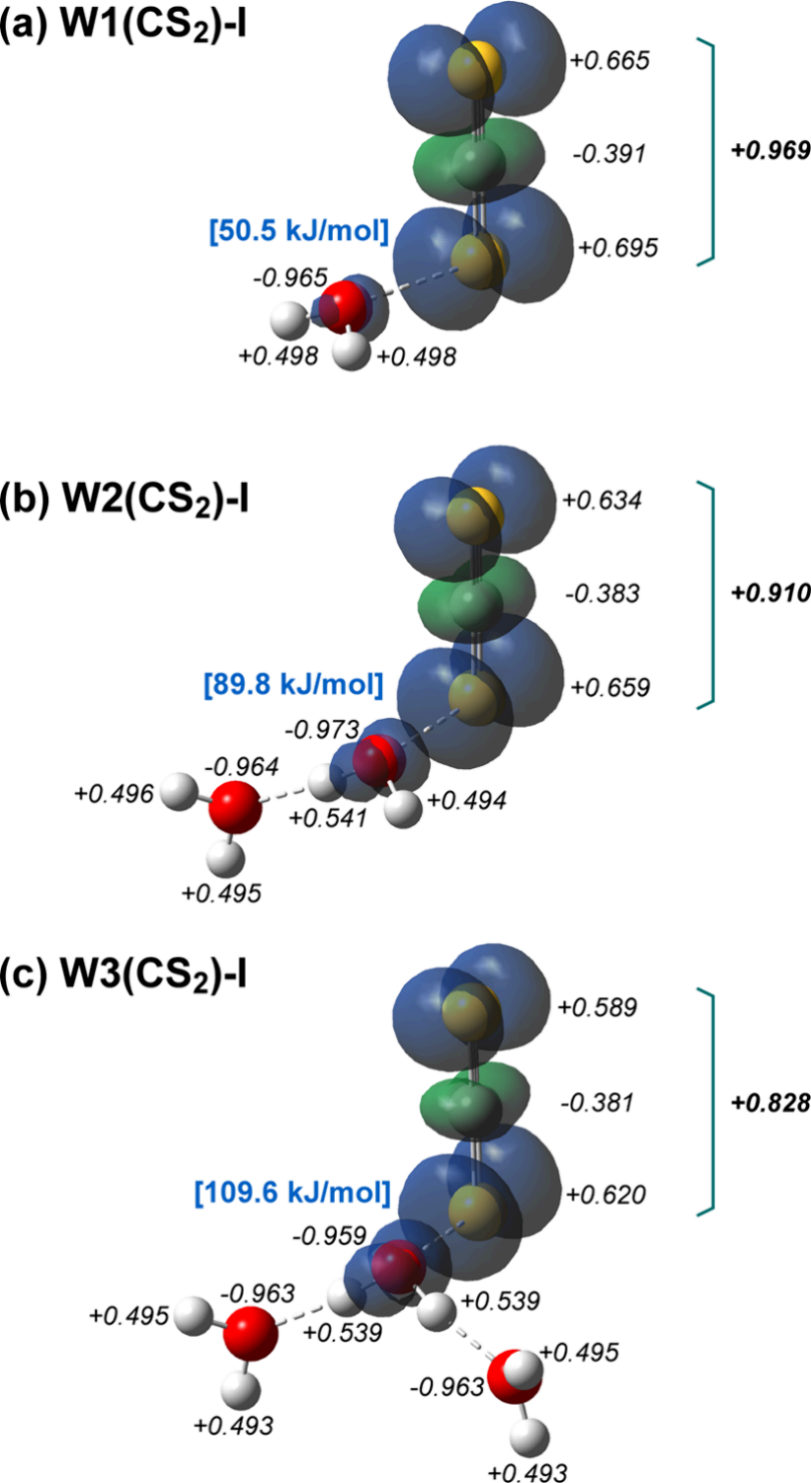
(a–c) Type-I structures of [H_2_O–CS_2_]^+^–(H_2_O)_*n*_ (*n* = 0–2), together with the NBO charges (black), spin density distributions, and the binding energies between the CS_2_–OH_2_ bond­(blue). The NBO charges were calculated at the CCSD­(T)/aug-cc-pVTZ level. The spin densities were calculated at the CCSD/aug-cc-pVDZ level, and the density is plotted at an isosurface value of 0.004. The binding energies were evaluated from BSSE-corrected electronic energies at the CCSD­(T)/aug-cc-pVTZ level with ZPE corrections performed at the CCSD/aug-cc-pVDZ level.

To further elucidate the distinct microhydration behaviors, it is instructive to compare the IPs of H_2_O and molecule X in more detail. Although the IP of O_2_ is lower than that of an isolated H_2_O molecule, additional hydration stabilizes the water moiety. For example, the IP of a water dimer (H_2_O)_2_ is 11.756 eV,^86^ which is lower than that of O_2_. This stabilization leads to an inversion of ionization preference upon microhydration, consistent with the observed dissociation of bare [H_2_O–O_2_]^+^–(H_2_O)_*n*_ (*n* = 1 and 2) via the O_2_ loss channel (see the SI). Despite this IP inversion, the positive charge remains preferentially localized on O_2_ rather than on water (see Figures 4(b) and (c)). If the positive charge were localized on water, a proton-transferred (type-III) structure would be expected, considering its strong preference in bare (H_2_O)_2_^+^. Nevertheless, because O_2_ has a low proton affinity (PA, 421 kJ/mol),^87^ such a proton-transferred configuration, where O_2_ acts as a proton acceptor for the H_3_O^+^ ion core, fails to sufficiently lower the total energy and is therefore energetically disfavored. As a result, the positive charge remains preferentially localized on O_2_, and the type-I structure is stabilized by a combination of charge-resonance and charge-(induced) dipole interactions. In contrast, for CS_2_, whose IP remains lower than that of water multimers, no such inversion occurs, and the positive charge remains localized on CS_2_ throughout microhydration. Notably, the ΔIP between CS_2_ and the water multimer decreases and enhances the charge-resonance effect with increasing microhydration.

Taken together, these results indicate that, in [H_2_O–X]^+^, localization of the positive charge on molecule X, which is favored by a low IP and/or a low PA, allows the hemibond to persist upon microhydration. On this basis, the previously reported disruption of the hemibond in [H_2_O–N_2_O]^+^ can be attributed to the relatively high IP of N_2_O (12.9 eV),^82^ which promotes charge localization on water and favors the proton-transferred structure.^79^

It is noteworthy that O_2_-containing clusters exhibit distinctive behavior, particularly in W3­(O_2_)-I, where approximately half of the positive charge is distributed over the water moiety. Such a charge distribution would, in principle, be expected to enhance charge-resonance interactions and thereby strengthen the O_2_–OH_2_ hemibond. Contrary to this expectation, however, the O_2_–OH_2_ binding energy of W3­(O_2_)-I is remarkably small (23.0 kJ/mol). This suggests that, even when the charge-resonance interaction is significant, O_2_ has an inherently low hemibonding capability, and the stabilization provided by the hemibond is insufficient to compensate for the concomitant weakening of the charge-(induced) dipole interaction upon microhydration.

To clarify whether this unexpectedly small binding energy indeed reflects a weak intrinsic hemibonding capability of O_2_, we quantitatively evaluate the binding energy of the O_2_–OH_2_ and CS_2_–OH_2_ hemibonds in [H_2_O–X]^+^ using an established theoretical expression^14,16,18^

1 $$D_{AB}≅\frac{D_{AA}+D_{BB}}{2}\exp\left(\frac{-\Delta IP}{2\sqrt{D_{AA}D_{BB}}}\right)$$

where *D*_AA_ and *D*_BB_ denote the binding energies of the hemibonded homodimer cations of molecules A and B, respectively, and ΔIP represents the IP difference between the two molecules. Details of the analysis are given in the SI. In this model, O_2_ or CS_2_ is treated as molecule A and H_2_O as molecule B. The hemibond binding energy *D*_AB_ depends not only on ΔIP between O_2_/CS_2_ and H_2_O but also on the binding energy of the homodimer cation *D*_AA_ (i.e., (O_2_)_2_^+^/(CS_2_)_2_^+^), which reflects intrinsic molecular properties such as the spatial overlap of lone-pair orbitals. In general, hemibonding interactions are expected to be stronger when ΔIP is smaller and/or *D*_AA_ is larger.

Table 1 summarizes the values of *D*_AA_ for O_2_ and CS_2_, together with the corresponding *D*_AB_ values for [H_2_O–X]^+^. The binding energy of the hemibonded (H_2_O)_2_^+^, *D*_BB_, was calculated to be 11949 cm^–1^. The optimized structures employed in the calculations of the homodimer cations are provided in Figure S5 in the SI. As summarized in Table 1, the homodimer hemibond binding energy *D*_AA_ of O_2_ (2982 cm^–1^) is substantially smaller than that of CS_2_ (6536 cm^–1^), indicating that O_2_ indeed possesses a markedly weaker intrinsic ability to form hemibonds. Nevertheless, the estimated heterodimer hemibond energy *D*_AB_ is larger for O_2_ (5144 cm^–1^) than for CS_2_ (2890 cm^–1^), owing to the smaller ΔIP between H_2_O and O_2_. This finding demonstrates that the evaluated intrinsic hemibond strength does not directly correspond to the total binding energy of [H_2_O–X]^+^. Instead, the observed binding energies reflect a balance between the intrinsic hemibonding interaction and nonorbital stabilizations, whose relative contributions depend sensitively on charge localization and its evolution upon microhydration.

**Table 1:** Binding Energies of the Hemibonded Homodimer Cation *D*_AA_ Calculated at the CCSD­(T)/aug-cc-pVTZ//CCSD/aug-cc-pVDZ Level of Theory, and Intrinsic Hemibond Binding Energies of the Hemibonded [H_2_O–X]^+^ Structures *D*_AB_ Estimated Using eq (1) for X = O_2_ and CS_2_

X	IP (eV)	*D*_AA_ (cm^–1^)	*D*_AB_ (cm^–1^)
O_2_	12.1	2982	5144
CS_2_	10.1	6536	2890

In the case of O_2_, the intrinsically weak hemibonding interaction cannot compensate for the substantial loss of electrostatic and induction stabilization accompanying charge delocalization, resulting in an overall decrease in the O_2_–OH_2_ binding energy. This reduction may render the hemibond susceptible to disruption upon further microhydration. In contrast, CS_2_ possesses a sufficiently strong intrinsic hemibonding capability, and no inversion of ionization preference occurs upon hydration, which results in the strengthening of the CS_2_–OH_2_ bond. Consequently, in such systems, the hemibond may remain intact even under dilute aqueous conditions.

In conclusion, we have examined hemibond formation in [H_2_O–X]^+^ (X = O_2_ and CS_2_) and its evolution upon microhydration by means of infrared photodissociation spectroscopy. The close agreement between experiment and theory enabled reliable structural assignments for [H_2_O–X]^+^–(H_2_O)_*n*_ (X = O_2_ and CS_2_; *n* = 0–2), demonstrating that the lowest-energy structures in all cases correspond to hemibonded (type-I) motifs. Analysis of NBO charges and spin density distributions revealed that the intermolecular binding is determined by a balance between charge-resonance (hemibonding) interactions and charge-(induced) dipole stabilization. Although microhydration enhances charge delocalization and thereby strengthens the charge-resonance component, it simultaneously reduces charge-(induced) dipole contributions through redistribution of the positive charge. The distinct trends observed for O_2_ and CS_2_ arise from differences in their intrinsic hemibonding capabilities and ionization potentials. On this basis, the present findings establish a consistent framework for predicting the stability of water-involving hemibonds under incipient solvation. These results bridge isolated gas-phase clusters and solvated environments, and provide molecular-level insight into the conditions under which hemibonds with water can persist in aqueous systems.

## Supplementary Material



## Data Availability

The data supporting this article are included in the Supporting Information.

## Notes

Experimental methods, calculational methods, IRPD spectra of [H_2_O–O_2_]^+^ and [H_2_O–CS_2_]^+^–N_2_ clusters, IRPD spectra of bare [H_2_O–O_2_]^+^–(H_2_O)_*n*_ (*n* = 1 and 2) clusters, IRPD spectra of N_2_-tagged [H_2_O–CS_2_]^+^–(H_2_O)_*n*_ (*n* = 1 and 2) clusters, optimized structures of the homodimer cations (O_2_)_2_^+^ and (CS_2_)_2_^+^, coordinates of the energy-optimized isomer structures of all isomers reported in this study, and complete author list of ref 85 (PDF)
