# Correction to “Microhydration Effects on Hemibonds in [H_2_O–X]^+^–(H_2_O)_*n*_ (X = O_2_ and CS_2_; *n* = 0–2): Infrared Spectroscopic Characterization toward Understanding Charge-Resonance Interactions in Aqueous Environments”

**DOI:** 10.1021/acs.jpclett.6c01046

**Published:** 2026-04-07

**Authors:** Tatsuki Hosoda, Mizuhiro Kominato, Asuka Fujii

**Affiliations:** Department of Chemistry, Graduate School of Science, 13101 Tohoku University, Sendai 980-8578, Japan

In the original version of the manuscript, Mizuhiro Kominato was inadvertently omitted from the author list. The corrected author list and the specific contributions of the added author are provided below.

The corresponding author (Fujii) regrets that at the time of submission, the foundational contributions made by Dr. Kominato during the conceptual stage of the research were not fully recognized due to an administrative misunderstanding. Dr. Kominato proposed the core research framework and provided essential theoretical predictions that guided the experimental direction. All authors, including the newly added author, have reviewed and agreed to this correction.

**Corrected Author List**: Tatsuki Hosoda, Mizuhiro Kominato, and Asuka Fujii*, Department of Chemistry, Graduate School of Science, Tohoku University, Sendai 980–8578, Japan.

**Revised Author Contributions**: Tatsuki Hosoda: Investigation (lead), Formal analysis, Data curation, Software (theoretical calculations), and Writing – original draft. Mizuhiro Kominato: Conceptualization, Methodology, and Formal analysis (initial theoretical predictions). Asuka Fujii: Conceptualization, Supervision, Funding acquisition, and Writing – review and editing.

