# Supplementary material for: Microhydration Effects on Hemibonds in [H2O–X]+–(H2O) n (X = O2 and CS2; n = 0–2): Infrared Spectroscopic Characterization toward Understanding Charge-Resonance Interactions in Aqueous Environments
Source: J Phys Chem Lett. 2026 Mar 23;17(13):3870–8. doi: 10.1021/acs.jpclett.6c00621 (PMC13051428; doi:10.1021/acs.jpclett.6c00621)
Supplement: Supplementary file 1 [file jz6c00621_si_001.pdf]

Supporting Information

Microhydration Effects on Hemibonds in $[H_2O{-}X]^+{-}(H_2O)_n$ ($X = O_2$ and CS_2; n = 0−2): Infrared Spectroscopic Characterization toward Understanding Charge-Resonance Interactions in Aqueous Environments

Tatsuki Hosoda and Asuka Fujii*

Department of Chemistry, Graduate School of Science, Tohoku University, Sendai 980-8578, Japan

* To whom correspondence addressed Email: asuka.fujii.c5@tohoku.ac.jp

Contents

1. **Methods**

   1.1 **Experimental** ········· **S2**

   1.2 **Calculational** ········· **S4**

2. **Additional Results**

   2.1 **IRPD spectra of $[H_2O{-}O_2]^+$ and $[H_2O{-}CS_2]^+{-}N_2$ clusters** ········· **S6**

   2.2 **IRPD spectra of bare $[H_2O{-}O_2]^+{-}(H_2O)_n$ (n = 1 and 2) clusters** ········· **S7**

   2.3 **IRPD spectra of N_2-tagged $[H_2O{-}CS_2]^+{-}(H_2O)_n$ (n = 1 and 2) clusters** ········· **S8**

   2.4 **Optimized structures of the homodimer cations $(O_2)_2^+$ and $(CS_2)_2^+$** ········· **S10**

3. **Coordinates of the energy-optimized isomer structures of all isomers reported in this study.** ········· **S11**

4. **Complete author lists of Reference 85** ········· **S16**

1. Methods

1.1 Experimental

Figure S1 shows a schematic diagram of the experimental apparatus. The experimental details have been described elsewhere.[1] Clusters were generated by electron ionization of a supersonic jet expansion of a gas mixture introduced into a vacuum chamber using a high-pressure pulsed valve (Even-Lavie valve[2]). (I) For O_2-containing clusters, a gaseous mixture of O_2 and Ar (5 and 95%) was used as a carrier gas. Water vapor was generated within both the in-line sample holder and the sample cartridge installed in the valve, allowing the carrier gas to pick up water molecules as it passed through these compartments. On the other hand, (II) for CS_2-containing clusters, a gaseous mixture of N_2 and Ar (5 and 95%) was used as a carrier gas. CS_2 vapor was generated in the sample holder, while H_2O vapor was generated in the sample cartridge, so that both CS_2 and H_2O were seeded into the carrier gas. In both (I) and (II), the carrier gas was expanded at a stagnation pressure of 70 atm. The resulting gas mixture was ionized by a 200 V electron beam generated from an electron gun (Omegatron Corporation) in the collisional region of the jet expansion, producing radical cation clusters via electron impact ionization. Produced ion clusters were introduced into a tandem-type quadrupole mass spectrometer through a skimmer. The cluster ions of interest were mass-selected in the first quadrupole and subsequently transferred into an octupole ion guide, where they were irradiated with infrared (IR) light. The IR radiation was generated by an optical parametric oscillator/amplifier (LaserVision) pumped by the fundamental output of a Nd:YAG laser (Spectra Physics, GCR 230). When the IR frequency was resonant with a vibrational transition of the cluster cation, vibrational predissociation occurred, producing fragment ions. The fragment ions were mass-selected in the second stage of the mass spectrometer and subsequently detected by a channel electron multiplier while scanning the IR frequency. The IR spectra of the size-selected cluster ions were obtained from the fragment ion yield as a function of IR frequency. The observed spectra were normalized to the IR laser power and calibrated to vacuum wavenumbers by referencing atmospheric water vapor absorption lines.

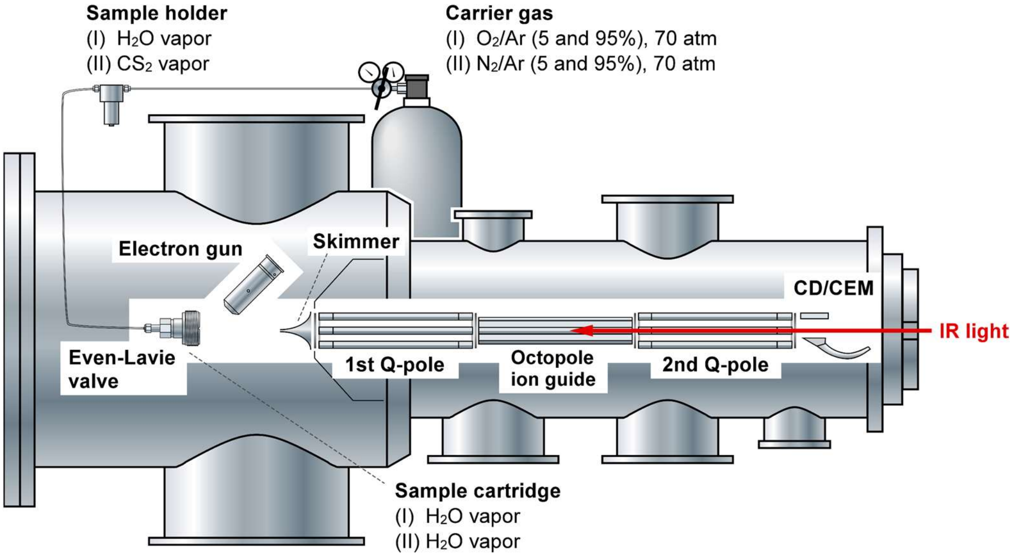

Figure S1. A schematic diagram of the experimental apparatus used in the present study. Q-pole = quadrupole mass spectrometer, CD = conversion dynode, CEM = channeltron electron multiplier. The carrier gases and the contents of the sample holder/cartridge are also shown: (I) O_2-containing clusters and (II) CS_2-containing clusters.

The O_2 loss channel was monitored for $[H_2O{-}O_2]^+{-}(H_2O)_n$ (n = 0−2), whereas the Ar loss channel was monitored for the Ar-tagged clusters. In both cases, the mass resolution of the 1st Q-mass spectrometer was set to Δm/z = ~1, which allowed the careful exclusion of the corresponding protonated species. For $[H_2O{-}CS_2]^+{-}(H_2O)_n$ (n = 0−2), the H_2O loss channel was monitored with a mass resolution of Δm/z = ~1. In addition, the N_2 loss channel was monitored for the N_2-tagged clusters. For the N_2-tagged species, while $[H_2O{-}CS_2]^+{-}N_2$ was produced with sufficient intensity to allow operation at high mass resolution (Δm/z = ~1), the signal intensity of $[H_2O{-}CS_2]^+{-}(H_2O)_n{-}N_2$ (n = 1 and 2) was insufficient to maintain this resolution. Consequently, the mass resolution was relaxed to Δm/z ≥ 2, and the radical cation clusters and the corresponding protonated species were detected simultaneously without mass separation (see Figure S4).

References

1. Mizuse, K.; Fujii, A. Infrared Photodisscoiation Spectroscopy of $H^+(H_2O)_6{\cdot}M_m$ (M=Ne, Ar, Kr, Xe, H_2, N_2, and CH_4): Messenger-Dependent Balance between H_3O^+ and $H_5O_2^+$ Core Isomers. *Phys. Chem. Chem. Phys.* **2011**, *13*, 7129−7135.
2. Even, U.; Jortner, J.; Noy, D.; Lavie, N.; Cossart-Magos, C. Cooling of Large Molecules below 1 K and He Clusters Formation. *J. Chem. Phys.* **2000**, *112*, 8068−8071.

1.2 Calculational

Energy-optimized structure searches of the clusters and harmonic vibrational frequency calculations were performed at the CCSD/aug-cc-pVDZ level of theory. All calculated vibrational frequencies were scaled by a factor of 0.951, which was determined by matching the calculated ν_3 frequency of the neutral water moiety in W3(O_2, Ar)-I to the corresponding experimental value. Spin density analyses were carried out at the same level, employing the density matrix formalism for coupled-cluster (CC) methods. For the optimized structures, single-point electronic energies and natural bond orbital (NBO) charges were computed at the CCSD(T)/aug-cc-pVTZ level to achieve improved energetic accuracy. Relative energies of the isomers were obtained by adding zero-point vibrational energy (ZPE) corrections, calculated at the CCSD/aug-cc-pVDZ level, to the single-point electronic energies evaluated at the CCSD(T)/aug-cc-pVTZ level. To obtain reliable binding energies for the clusters shown in Figures 4 and 5, basis set superposition error (BSSE) corrections were applied using the counterpoise method.

For O_2-containing clusters, explicit consideration of spin multiplicity is essential. O_2 is an open-shell triplet species; therefore, two possible spin states arise in the O_2-water radical cation clusters. One corresponds to a doublet state arising from O_2^+ interacting with $(H_2O)_n$, whereas the other corresponds to a quartet state arising from neutral O_2 interacting with $(H_2O)_n^+$. Calculations were performed for both multiplicities; however, no stable type-I (hemibonded) structure was obtained for the quartet state. Accordingly, only the doublet spin state is considered in the present study. For the CS_2-containing clusters, all calculations were carried out in the doublet spin state.

We also evaluate the binding energy of the hemibond using a theoretical expression.[1-3] The binding energy of a hemibond formed between molecules A and B, D_{AB} can be expressed as

$$D_{AB} \cong \frac{D_{AA} + D_{BB}}{2} exp\left(\frac{-\Delta IP}{2\sqrt{D_{AA}D_{BB}}}\right) \quad (1)$$

where D_{AA} and D_{BB} denote the binding energies of the hemibonded homodimer cations of molecules A and B, respectively, and ΔIP represents the IP difference between the two molecules. The values of D_{AA} for $(O_2)_2^+$ and $(CS_2)_2^+$ and D_{BB} for $(H_2O)_2^+$ were computed at the CCSD(T)/aug-cc-pVTZ//CCSD/aug-cc-pVDZ level without BSSE correction. These values were then substituted into eq. (1) to estimate D_{AB} for $[H_2O{-}X]^+$ (X = O_2 and CS_2). The optimized structures of the isomers used for the D_{AA} calculations are shown in Figure S5. The validity of this approach is supported by previous studies. For example, in $[H_2O{-}CO_2]^+$, a hydrogen-bonded structure was observed instead of a hemibonded one despite the small ΔIP between H_2O and CO_2 (ΔIP ~1.2 eV).[4] This behavior was attributed to the small value of D_{AA} for CO_2 (4198 cm^{-1}), indicating that the weak intrinsic hemibonding capability of CO_2 suppresses hemibond formation even when ΔIP is comparable to that of other hemibonded systems.

References

1. Clark, T. Odd-Electron σ Bonds. *J. Am. Chem. Soc.* **1988**, *110*, 1672−1678.
2. Hiberty, P. C.; Humbel, S.; Archirel, P. Nature of the Differential Electron Correlation in Three-Electron Bond Dissociations. Efficiency of a Simple Two-Configuration Valence Bond Method with Breathing Orbitals. *J. Phys. Chem.* **1994**, *98*, 11697−11704.
3. Clark, T. Odd-Electron Bonds. *ChemPhysChem* **2017**, *18*, 2766−2771.
4. Kominato, M.; Fujii, A. Infrared Spectroscopy of $[H_2O–X_n]^+$ (n = 1–3, X = N_2, CO_2, CO, and N_2O) Radical Cation Clusters: Competition between Hydrogen Bond and Hemibond Formation of the Water Radical Cation. *Phys. Chem. Chem. Phys.* **2023**, *25*, 14726−14735.

2. Additional Results

2.1 IRPD spectra of $[H_2O{-}O_2]^+$ and $[H_2O{-}CS_2]^+{-}N_2$ clusters

Figure S2(a) shows the observed infrared spectrum of $[H_2O{-}O_2]^+$, while Figure S2(b) presents that of $[H_2O{-}CS_2]^+{-}N_2$, together with the optimized stable structures and their corresponding calculated spectra. In the observed spectrum of $[H_2O{-}O_2]^+$, a prominent band appears at 3470 cm^{-1}. This band is assigned to the ν_1 vibration of the H_2O moiety based on comparison with the calculated spectrum. In contrast, the ν_3 band is not clearly observed and appears significantly weakened and broadened around 3550 cm^{-1}. This behavior can be attributed to internal rotational motion of the H_2O moiety. The ν_3 vibration forms a perpendicular band, meaning that its transition dipole moment is oriented perpendicular to the principal axis of internal rotation and the mode corresponds to transitions with $\Delta k = \pm 1$ (where k is the internal rotational quantum number associated with the rotation of the H_2O moiety). For perpendicular bands, the selection rules are $\Delta J = 0, \pm 1$ and $\Delta k = \pm 1$. Since transitions arise from multiple thermally populated k levels, significant overlap of rovibrational lines occurs, and the intense Q branch ($\Delta J = 0$) further contributes to the apparently broadened band shape.

In the observed spectrum of $[H_2O{-}CS_2]^+{-}N_2$, two distinct bands are detected at approximately 3520 and 3650 cm^{-1}. These bands are assigned to the N_2-bound OH stretching and free OH stretching vibrations, respectively, as calculated for the W1(CS_2, N_2)-I structure. Weaker features near 3150 and 3600 cm^{-1} are attributed to the bending overtone and combination band.

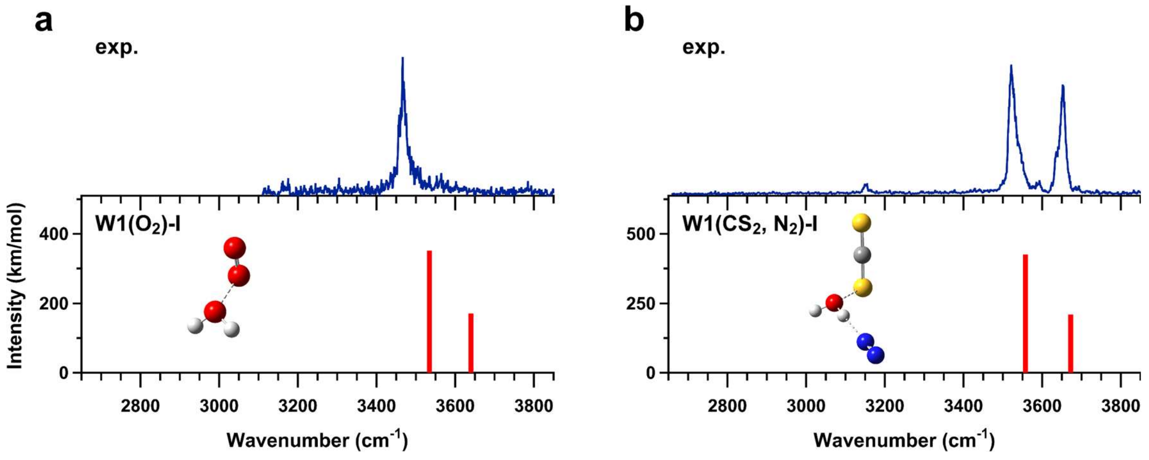

Figure S2. Comparison of the observed IR spectra of (a) $[H_2O{-}O_2]^+$ and (b) $[H_2O{-}CS_2]^+{-}N_2$ with the calculated spectra. The calculated spectra are scaled by a factor of 0.951. The corresponding optimized structures are also shown.

2.2 IRPD spectra of $[H_2O-O_2]^+-(H_2O)_n$ (*n* = 1 and 2) clusters

Figures S3(a) and (b) show comparisons between the observed and calculated IR spectra of $[H_2O-O_2]^+-H_2O$ and $[H_2O-O_2]^+-(H_2O)_2$, respectively. In Figure S3(a), four prominent bands are experimentally observed: a broad band around 2750 cm^{-1}, a strong band near 3500 cm^{-1}, and two bands above 3600 cm^{-1}. According to the calculated spectrum of the most stable isomer, W2(O_2)-I, these bands are assigned, respectively, to the OH stretching vibrations of the hydrogen-bonded OH group, the dangling OH stretching vibration, and the ν_1 and ν_3 modes of the neutral H_2O moiety. Similarly, in Figure S3(b), three prominent bands are observed at approximately 2700, 3640, and 3730 cm^{-1}, together with a weaker band around 3550 cm^{-1}. These bands are assigned consistently with the discussion in the main text (*i.e.*, the overlapping contributions of W3(O_2)-I and W3(O_2)-III).

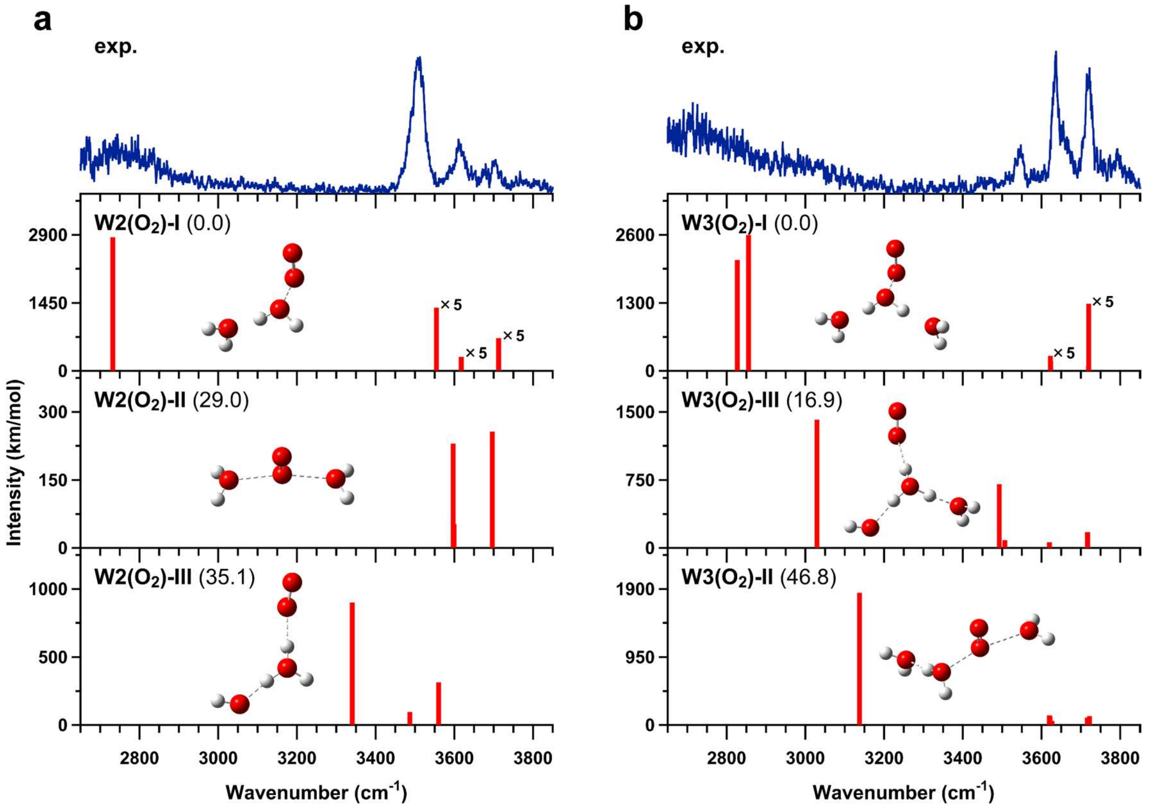

Figure S3. Comparison of the observed IR spectra of (a) $[H_2O-O_2]^+-H_2O$ and (b) $[H_2O-O_2]^+-(H_2O)_2$ with the calculated spectra. The corresponding optimized structures are also shown. The calculated spectra are scaled by a factor of 0.951. The values given in parentheses indicate ZPE corrected relative energies (in kJ/mol)

2.3 IRPD spectra of N_2-tagged $[H_2O{-}CS_2]^{+}{-}(H_2O)_n$ (n = 1 and 2) clusters

Figures S4(a) and (b) show comparisons between the observed and calculated IR spectra of $[H_2O{-}CS_2]^{+}{-}H_2O{-}N_2$ and $[H_2O{-}CS_2]^{+}{-}(H_2O)_2{-}N_2$, respectively. As described in the Experimental section, the signal intensities of $[H_2O{-}CS_2]^{+}{-}(H_2O)_n{-}N_2$ (n = 1 and 2) were insufficient to distinguish the radical cation from the corresponding protonated species using our mass spectrometer; therefore, both species were detected simultaneously. In Figure S4(a), the simulated spectrum of W2(CS_2, N_2)-I, the most stable isomer of $[H_2O{-}CS_2]^{+}{-}H_2O{-}N_2$, is presented along with that of the protonated counterpart W2(CS_2, N_2)-protonated. In the observed spectrum of $[H_2O{-}CS_2]^{+}{-}H_2O{-}N_2$, six prominent bands are observed at approximately 2900, 3170, 3400, 3520, 3640, and 3720 cm^{-1}. The experimental spectrum is satisfactorily reproduced by the combination of the calculated spectra of W2(CS_2, N_2)-I and W2(CS_2, N_2)-protonated, and the corresponding band assignments are indicated by dotted lines. The band at 3520 cm^{-1} is assigned to the N_2-bound OH stretching vibration of W2(CS_2, N_2)-I, and the ν_1 and ν_3 bands of the neutral H_2O moiety are also clearly identified. Therefore, the overlap between the dangling OH stretching vibration and the ν_1 mode of the terminal H_2O moiety observed at approximately 3630 cm^{-1} in the bare $[H_2O{-}CS_2]^{+}{-}H_2O$ cluster is resolved by employing N_2-tagging, thereby supporting the discussion presented in the main text.

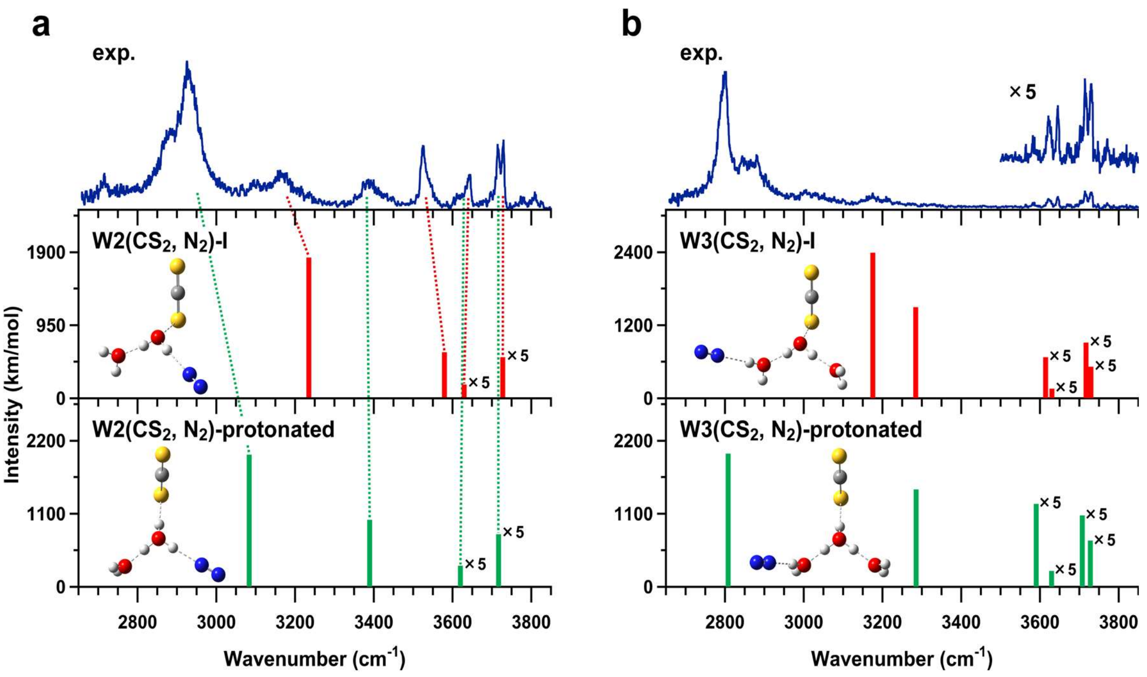

Figure S4. Comparison between the observed IR spectra of (a) $[H_2O{-}CS_2]^{+}{-}H_2O{-}N_2$ and (b) $[H_2O{-}CS_2]^{+}{-}(H_2O)_2{-}N_2$ and their calculated spectra for the most stable radical cation isomers. Also included are the calculated spectra of the protonated counterparts along with their optimized structures. The calculated spectra are scaled by a factor of 0.951. The values given in parentheses indicate ZPE corrected relative energies (in kJ/mol).

In Figure S4(b), the simulated spectra of W3(CS_2, N_2)-I and W3(CS_2, N_2)-protonated are presented. The experimental spectrum was not satisfactorily reproduced by the two calculated spectra. In the free OH stretching region, multiple bands were observed; however, these bands are weak in intensity, and the corresponding calculated spectra become increasingly complex as the number of water molecules increases in the cluster, making reliable assignment difficult. Furthermore, a very intense band appears at approximately 2800 cm^{-1}. Although the calculated spectrum of W3(CS_2, N_2)-protonated predicts an OH stretching band of H_3O^+ hydrogen-bonded to H_2O around 2800 cm^{-1}, consistent with the observed position, the experimentally observed band is unusually sharp. Given that hydrogen-bonded OH stretching vibrations are typically broad, this assignment remains tentative. Therefore, in the present study, we concentrate on the results of the bare $[H_2O-CS_2]^+-(H_2O)_2$ cluster alone.

2.4 Optimized structures of the homodimer cations $(O_2)_2^+$ and $(CS_2)_2^+$

Figures S5(a) and (b) show the optimized structures of $(O_2)_2^+$ and $(CS_2)_2^+$, respectively, together with their spin states, relative energies (including zero-point energy corrections), and $D_{AA/AB}$ values as defined in the main text. Geometry optimizations were performed at the CCSD/aug-cc-pVDZ level of theory, followed by single-point energy calculations at the CCSD(T)/aug-cc-pVTZ level. In the search for stable isomers, we referred to the studies of Watabe *et al.*[1] for $(O_2)_2^+$ and Koyama *et al.*[2] for $(CS_2)_2^+$. It should be noted that the computational levels employed in the present study differ from those used in the previous reports; consequently, the energetic ordering of the isomers is not identical. For $(O_2)_2^+$, three structures denoted as a1, a2, and a3 were obtained, whereas for $(CS_2)_2^+$, two structures denoted as b1 and b2 were identified. The dimer binding energies, D_{AA}, were calculated for these structures, and the corresponding D_{AB} values, representing the binding energies with H_2O, were subsequently evaluated. In the main text, the $D_{AA/AB}$ values of the a2 and b2 structures, which exhibit pronounced orbital interaction character, are presented.

	**a $(O_2)_2^+$**			**b $(CS_2)_2^+$**	
**spin**	quartet	quartet	doublet	doublet	doublet
**structure**	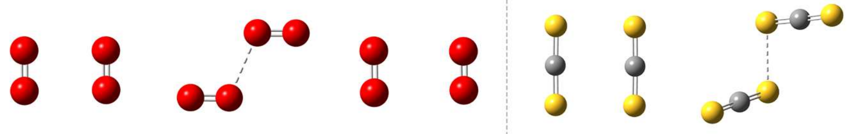				
	**a1**	**a2**	**a3**	**b1**	**b2**
**relative energy (cm^{-1})**	0	+91	+1995	0	+541
**D_{AA} (cm^{-1})**	3073	2982	1078	7078	6536
**D_{AB} (cm^{-1})**	5204	5144	3505	3112	2890

Figure S5. Optimized structures of (a) $(O_2)_2^+$ and (b) $(CS_2)_2^+$, together with their spin states, ZPE corrected relative energies, and $D_{AA/AB}$ values.

References

1. Watabe, Y.; Koshiba, T.; Ito, Y.; Kanno, M.; Ohshimo, K.; Misaizu, F. UV Photodissociation of Oxygen Dimer Cation O_4^+ Studied by an Ion Imaging Technique and Theoretical Calculations. *J. Chem. Phys.* **2025**, *163*, 024301.
2. Koyama, M.; Muramatsu, S.; Hirokawa, Y.; Iriguchi, J.; Matsuyama, A.; Inokuchi, Y. Correlation of the Charge Resonance Interaction with Cluster Conformations Probed by Electronic Spectroscopy of Dimer Radical Cations of CO_2 and CS_2 in a Cryogenic Ion Trap. *J. Phys. Chem. Lett.* **2024**, *15*, 1493–1499.

3. **Coordinates of the energy-optimized isomers structures of all isomers reported in this study.**

O_2-containing clusters

W1(O_2)-I

O	-0.075018	-0.923321	-0.281769
H	0.830923	-0.924346	0.072077
H	-0.499188	-0.141350	0.111345
O	-1.367161	-3.075727	-1.037748
O	-1.123344	-2.714185	0.008735

W2(O_2)-I

O	1.140336	-0.094407	0.169847
H	1.909246	0.479729	0.345663
H	0.340380	0.294166	0.665747
O	1.498751	-1.719774	1.090026
O	1.341501	-2.598720	0.353985
O	-0.999881	0.706373	1.344004
H	-1.761225	0.949773	0.800026
H	-1.077363	1.228947	2.153226

W2(O_2)-II

O	0.018788	0.408790	-2.253779
H	-0.718922	0.820690	-2.725889
H	0.806419	0.714704	-2.725817
O	-0.004609	0.073498	-0.000063
O	-0.082659	-1.049526	-0.000319
O	0.018585	0.407775	2.253805
H	-0.719168	0.819463	2.726034
H	0.806173	0.713477	2.726052

W1(O_2, Ar)-I

O	0.189140	-0.911875	-0.337647
H	0.736981	-0.915765	-0.031967
H	-0.610905	-0.169896	0.129758
O	-1.440133	-3.090850	-1.030335
O	-1.130473	-2.719569	-0.001929
Ar	3.095436	-1.129003	0.578715

W2(O_2, Ar)-I

O	1.102434	-0.109399	0.108718
H	1.882049	0.467853	0.234241
H	0.324986	0.289985	0.632134
O	1.522114	-1.667634	1.082500
O	1.359957	-2.589872	0.396973
O	-0.979369	0.714035	1.366796
H	-1.777812	0.910192	0.858115
H	-1.018297	1.282513	2.147305
Ar	3.971021	1.687598	0.369698

W2(O_2, Ar)-II

O	-0.003631	0.420747	-2.257255
H	-0.740274	0.858639	-2.706579
H	0.777458	0.688834	-2.761566
O	0.038192	0.055934	0.009933
O	-0.086513	-1.063048	-0.015238
O	0.157300	0.360884	2.235567
H	-0.556694	0.810167	2.712463
H	0.968158	0.668402	2.665495
Ar	-2.685439	1.794137	3.736953

W2(O_2)-III

O	-0.051966	-0.758508	-0.466580
H	0.813918	-1.186230	-0.647343
H	-0.243159	0.310052	0.618979
O	-0.278874	0.333572	3.983710
O	0.346581	0.939873	4.824138
O	-0.454683	1.087113	1.263554
H	-0.387360	0.832122	2.212899
H	-1.332019	1.472627	1.082656

W3(O_2)-I

O	-0.000251	-0.177753	0.595885
H	0.831990	-0.658763	0.276442
H	-0.833660	-0.656812	0.276543
O	0.001353	1.274326	-0.493358
O	0.002577	2.253846	0.156293
O	-2.261743	-1.204611	-0.126464
H	-2.934870	-1.358532	0.549650
H	-2.461671	-1.821582	-0.842078
O	2.258751	-1.209960	-0.126666
H	2.931505	-1.365660	0.549411
H	2.457285	-1.827129	-0.842497

W3(O_2)-II

O	0.156477	0.439482	-2.365634
H	-0.615120	0.976269	-2.689880
H	0.956853	0.935875	-2.598403
O	-0.057073	0.157681	-0.346324
O	-0.283334	-0.949447	-0.220609
O	-0.099484	0.474427	2.093414

W2(O_2, Ar)-III

O	-0.079142	-0.735347	-0.481767
H	0.765640	-1.207280	-0.650047
H	-0.245811	0.349213	0.620256
O	-0.319125	0.353321	3.989617
O	0.343102	0.915044	4.832775
O	-0.436622	1.128990	1.258362
H	-0.388858	0.866282	2.205215
H	-1.304293	1.543436	1.067233
Ar	-3.276856	2.570336	0.586965

W3(O_2, Ar)-I

O	-0.005939	-0.133002	0.689797
H	0.802532	-0.569134	0.261907
H	-0.853062	-0.411272	0.208260
O	0.175567	1.570725	0.101538
O	0.215657	2.304001	1.020271
O	-2.285522	-0.681804	-0.397110
H	-3.007446	-1.007292	0.156629
H	-2.467649	-1.006269	-1.288213
O	2.194023	-1.108920	-0.251936
H	2.804577	-1.561115	0.345132
H	2.368395	-1.467758	-1.131335
Ar	0.118922	-0.040902	-2.907917

W3(O_2, Ar)-II

O	0.114527	0.435212	-2.382689
H	-0.667479	0.971039	-2.685786
H	0.909396	0.955143	-2.587837
O	-0.077605	0.156788	-0.383600
O	-0.272674	-0.956398	-0.240330
O	-0.107858	0.489705	2.074272

H	-0.821044	0.847316	2.616737
H	0.672066	0.555850	2.669421
O	-2.081790	1.660247	-3.122870
H	-2.580762	1.284961	-3.860120
H	-2.271571	2.606827	-3.145587

H	-0.837892	0.831620	2.606721
H	0.663409	0.586378	2.648088
O	-2.142944	1.643376	-3.084707
H	-2.652252	1.266709	-3.814156
H	-2.339268	2.588764	-3.100887
Ar	3.204582	1.946282	-2.947852

W3(O_2)-III

O	2.123805	-2.126934	-0.175712
H	2.003557	-3.067843	0.073195
H	1.102550	-0.892131	0.239757
O	-2.158028	-0.116976	-0.459030
O	-3.131802	-0.001069	0.250083
O	0.606345	-0.061609	0.497692
H	-0.317566	-0.088293	0.182852
H	1.085835	0.810759	0.202268
O	1.682076	2.101114	-0.130910
H	2.031242	2.694786	0.547232
H	2.157890	2.295830	-0.948967

W3(O_2, Ar)-III

O	2.127267	-2.136795	-0.180389
H	2.002349	-3.078887	0.061168
H	1.104583	-0.894108	0.235723
O	-2.163216	-0.108175	-0.446931
O	-3.130350	0.023955	0.268404
O	0.613586	-0.061868	0.492918
H	-0.311816	-0.088282	0.183841
H	1.096143	0.813790	0.190807
O	1.681305	2.087422	-0.146900
H	2.038682	2.675796	0.533600
H	2.170037	2.264174	-0.961205
Ar	2.830322	4.176996	2.383508

CS_2-containing clusters

W1(CS_2)-I

O	1.036252	0.014510	0.250303
H	1.672075	0.736893	0.145857
H	0.198523	0.453704	0.456048
C	1.596412	-3.015477	0.111790
S	1.717876	-2.173850	1.454214
S	1.473423	-3.832334	-1.217336

W1(CS_2, N_2)-I

O	1.030756	-0.154392	0.250818
H	1.719223	0.528966	0.298009
H	0.199997	0.312946	0.420055
C	1.437298	-3.148022	0.096307
S	1.445601	-2.326085	1.460585
S	1.426995	-3.942184	-1.247964
N	4.123076	2.744284	0.282756
N	3.327401	1.966331	0.313751

W2(CS_2)-I

O	1.081636	-0.030000	0.186011
H	1.764110	0.655509	0.129567
H	0.308898	0.385157	0.641832
O	-1.136711	0.910683	1.402194
H	-1.909161	1.145767	0.872645
H	-1.196070	1.463597	2.191315
C	1.574477	-2.937957	0.087653
S	1.778236	-1.986742	1.354902
S	1.373553	-3.859924	-1.150452

W2(CS_2)-II

O	0.949416	0.292585	-0.049740
H	1.578004	0.987737	-0.287456
H	0.096063	0.745167	-0.007135
O	2.253880	-3.932205	3.064341
H	1.653632	-4.309877	3.721559
H	3.135190	-4.068864	3.437804
C	1.518086	-2.760443	0.265400
S	1.603025	-1.802226	1.530927
S	1.433787	-3.701116	-0.978227

W3(CS_2)-I

O	0.970947	0.127399	0.155680
H	1.679375	0.814333	0.201245
H	0.185162	0.461721	0.652240
O	-1.303381	0.798454	1.463325
H	-2.113277	0.912464	0.950147

W2(CS_2, N_2)-I

O	1.059276	-0.018381	0.249245
H	1.770435	0.641954	0.305876
H	0.266155	0.375503	0.688978
O	-1.220629	0.852193	1.399724
H	-1.970515	1.091228	0.840332
H	-1.330488	1.375483	2.203465
C	1.539317	-2.902390	0.081943
S	1.642559	-1.980616	1.384208
S	1.437079	-3.795131	-1.187811
N	4.326518	2.668707	0.153868
N	3.474572	1.958299	0.247428

W2(CS_2, N_2)-protonated

O	-0.039531	-0.610311	-0.411801
H	0.820164	-0.701087	-0.844145
H	-0.260327	0.359996	0.670416
O	-0.383191	1.110526	1.373573
H	-0.550788	0.751518	2.288535
H	-1.097866	1.735643	1.122594
C	0.282114	0.771684	4.916589
S	-0.954397	-0.013567	4.264073
S	1.468736	1.516145	5.589332
H	-0.478903	-1.468126	-0.479992
N	-2.414311	3.056830	0.641413
N	-3.154029	3.841876	0.368862

W3(CS_2, N_2)-I

O	0.958709	0.160892	0.162661
H	1.703352	0.807853	0.214495
H	0.197144	0.522583	0.683526
O	-1.203357	0.890183	1.572314
H	-2.056288	0.958205	1.120796

H	-1.430417	1.336720	2.254354
C	1.614305	-2.705611	0.076034
S	1.741014	-1.678709	1.294245
S	1.489503	-3.701685	-1.112157
O	3.075412	1.831313	0.142403
H	3.363413	2.469003	0.807238
H	3.362168	2.202638	-0.701500

H	-1.258358	1.506586	2.312400
C	1.601760	-2.669109	0.031280
S	1.630784	-1.681106	1.286998
S	1.571527	-3.627150	-1.193974
O	3.145219	1.760474	0.143470
H	3.513852	2.325041	0.834034
H	3.400260	2.181389	-0.687188
N	-3.986389	1.022380	0.044861
N	-4.966034	1.053119	-0.483158

W3(CS_2)-II

O	0.930100	0.222321	-0.072389
H	1.529603	0.932945	-0.335947
H	0.053735	0.628030	-0.108397
O	2.230685	-3.874907	3.046707
H	1.610533	-4.153743	3.734428
H	3.103403	-3.792784	3.490481
C	1.599291	-2.753768	0.356090
S	1.621850	-1.881856	1.691037
S	1.576961	-3.602939	-0.948102
O	4.756400	-3.606706	4.085585
H	5.388533	-4.332254	4.009354
H	5.023363	-3.127214	4.879469

W3(CS_2, N_2)-protonated

O	-0.022222	-0.657589	-0.289927
H	0.857152	-0.814458	-0.665803
H	-0.268715	0.393457	0.747841
O	-0.396166	1.166566	1.409456
H	-0.599182	0.835661	2.315551
H	-1.090818	1.837916	1.101752
C	0.268026	0.686222	4.973404
S	-1.075058	0.062286	4.371225
S	1.567006	1.281008	5.592935
H	-0.508605	-1.488004	-0.360117
O	-2.057930	2.910174	0.555825
H	-1.801476	3.834895	0.447712
H	-3.020316	2.900849	0.630949
N	3.776777	-1.190186	-2.016895
N	2.781993	-1.060178	-1.534713

Homodimer cation

a1

O	-2.523964	0.259873	-0.014036
O	-3.671207	0.249806	-0.012985
O	-2.487787	-2.104375	0.233023

b1

C	-0.763909	-3.021331	0.515074
S	-0.613403	-2.303800	1.909683
S	-0.875559	-3.737523	-0.883868

O	-3.665507	-2.114676	0.234099

a2

O	-2.815454	-0.197772	0.133285
O	-3.954943	-0.421002	0.225304
O	-4.360846	-2.369970	1.028719
O	-5.500333	-2.593206	1.120744

a3

O	-2.536590	0.360825	-0.024585
O	-3.660490	0.351176	-0.023578
O	-2.472272	-2.205535	0.243594
O	-3.679112	-2.215838	0.244670

C	2.433964	-2.911068	0.158169
S	2.283432	-3.628573	-1.236452
S	2.545644	-2.194905	1.557119

b2

C	-0.565659	-2.930114	0.601246
S	-0.418538	-3.266771	2.169005
S	-0.742703	-2.621615	-0.906882
C	2.292424	-1.420165	3.022023
S	2.310436	-2.902696	2.393770
S	2.312280	0.002551	3.635532

4. Complete author lists of Reference 85

85. Frisch, M. J.; Trucks, G. W.; Schlegel, H. B.; Scuseria, G. E.; Robb, M. A.; Cheeseman, J. R.; Scalmani, G.; Barone, V.; Petersson, G. A.; Nakatsuji, H.; Li, X.; Caricato, M.; Marenich, A. V.; Bloino, J.; Janesko, B. G.; Gomperts, R.; Mennucci, B.; Hratchian, H. P.; Ortiz, J. V.; Izmaylov, A. F.; Sonnenberg, J. L.; Williams-Young, D.; Ding, F.; Lipparini, F.; Egidi, F.; Goings, J.; Peng, B.; Petrone, A.; Henderson, T.; Ranasinghe, D.; Zakrzewski, V. G.; Gao, J.; Rega, N.; Zheng, G.; Liang, W.; Hada, M.; Ehara, M.; Toyota, K.; Fukuda, R.; Hasegawa, J.; Ishida, M.; Nakajima, T.; Honda, Y.; Kitao, O.; Nakai, H.; Vreven, T.; Throssell, K.; Montgomery, J. A., Jr.; Peralta, J. E.; Ogliaro, F.; Bearpark, M. J.; Heyd, J. J.; Brothers, E. N.; Kudin, K. N.; Staroverov, V. N.; Keith, T. A.; Kobayashi, R.; Normand, J.; Raghavachari, K.; Rendell, A. P.; Burant, J. C.; Iyengar, S. S.; Tomasi, J.; Cossi, M.; Millam, J. M.; Klene, M.; Adamo, C.; Cammi, R.; Ochterski, J. W.; Martin, R. L.; Morokuma, K.; Farkas, O.; Foresman, J. B.; Fox, D. J. *Gaussian 16*, Revision A.03, Gaussian, Inc., Wallingford, CT, 2016.
